# The complex nature of oestrogen signalling in breast cancer: enemy or ally?

**DOI:** 10.1042/BSR20160017

**Published:** 2016-06-30

**Authors:** Yulia Lipovka, John P. Konhilas

**Affiliations:** * Department of Physiology, Sarver Molecular Cardiovascular Research Program, University of Arizona, Tucson, AZ 85724, U.S.A.

**Keywords:** breast cancer, 17-β-oestradiol, oestrogen, oestrogen receptors

## Abstract

The pleiotropic nature of oestradiol, the main oestrogen found in women, has been well described in the literature. Oestradiol is positioned to play a unique role since it can respond to environmental, genetic and non-genetic cues to affect genetic expression and cellular signalling. In breast cancer, oestradiol signalling has a dual effect, promoting or inhibiting cancer growth. The potential impact of oestradiol on tumorigenesis depends on the molecular and cellular characteristics of the breast cancer cell. In this review, we provide a broad survey discussing the cellular and molecular consequences of oestrogen signalling in breast cancer. First, we review the structure of the classical oestrogen receptors and resultant transcriptional (genomic) and non-transcriptional (non-genomic) signalling. We then discuss the nature of oestradiol signalling in breast cancer including the specific receptors that initiate these signalling cascades as well as potential outcomes, such as cancer growth, proliferation and angiogenesis. Finally, we examine cellular and molecular mechanisms underlying the dimorphic effect of oestrogen signalling in breast cancer.

## OESTRADIOL AND ITS SIGNALLING

Oestrogens are steroid hormones that play key roles in growth, development, reproduction and maintenance of a diverse range of mammalian tissues. The three most common oestrogens are oestrone (E1), 17β-oestradiol (E2) and oestriol (E3). Oestrone and oestradiol are synthesized by the aromatization of androstenedione and testosterone respectively. Oestriol is synthesized from oestrone via a 16α-hydroxyoestrone intermediate [1]. Oestradiol is the predominant oestrogen during the premenopausal period. After menopause, oestrone is the main oestrogen. In premenopausal women, ovaries constitute the primary biosynthetic source of oestrogens. Oestrogen is also synthesized in extragonadal tissues including mesenchymal cells of the adipose tissue including that of the breast, osteoblasts and chondrocytes, aortic smooth muscle cells and vascular endothelium, as well as numerous parts of the brain [2].

### Oestrogen receptor structure

The physiological actions of oestradiol are mediated primarily through the classical oestrogen receptors (ER), ERα and ERβ. ERs are members of the nuclear hormone receptor (NHR) family and are composed of several functional domains. Spanning from NH2- to COO-terminus, the main functional domains of ER are the N-terminal domain (NTD), DNA-binding domain (DBD) and ligand-binding domain (LBD). The LDB consists of 11 α-helices and contains the hormone binding pocket, co-regulator interaction sites and homo- or heterodimerization interface. The DBD domain binds to the oestrogen response elements (EREs), which reside near the promoter or enhancer regions, and modulate recruitment of co-activators [3]. Two activation function (AF) domains, AF1 and AF2, located within the NTD and LBD respectively, are responsible for regulating the transcriptional activity of ER. AF1 function is hormone-independent, whereas AF2 requires hormone presence to become activated [4]. A ‘hinge region’, localized next to DBD, contains the nuclear localization signal, which gets exposed upon ligand binding [5]. The C-terminal portion of the receptor modulates gene transcription in a ligand-specific manner and affects dimerization [6,7].

ERα and ERβ are encoded by two different genes located on different chromosomes (locus 6q25.1 and locus 14q23-24.1 respectively) [8,9]. The wild type receptors (ERα-66 and ERβ1) share high degree of homology in the DBD (∼96% amino acid identity) and LBD (∼58% amino acid identity). The NTD region of ERβ is shorter than that of ERα and only shares ∼15% of sequence homology. The two receptors also differ in the composition of their hinge region and the C-terminal domain [10,11]. In addition to the wild type ER, there are multiple variant isoforms that originate by protein truncation or single amino acid mutations. The most referred ERα isoforms are ERα-46 and ERα-36. ERα-46 is a truncated variant that lacks the transcriptional activation domain AF1 [12]. ERα-36 lacks both AF1 and AF2 and has partial dimerization and LBDs. With three myriostoylation sites within its structure, this last receptor isoform is usually located to the plasma membrane [13].

**Figure 1:**
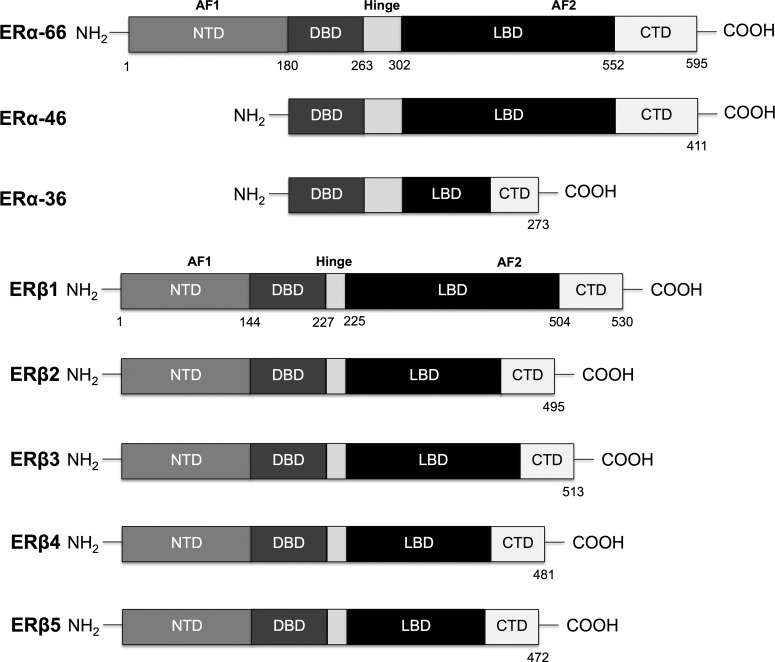
Primary structure of the classical oestrogen receptors Schematic representation of the functional domains composing full length ERα (ERα-66) and ERβ (ERβ1) and their most commonly referred isoforms. ERs are composed of NTD, DBD, hinge region, LBD and C-terminal domain (CTD).

ERβ has multiple isoforms resulting from alternative splicing of the last coding exon (exon 8) (ERβ2, ERβ3, ERβ4 and ERβ5) [14]. These isoforms diverge in their LBD. A more recent study showed that only the wild type variant (ERβ1) is fully functional, whereas ERβ2, ERβ4 and ERβ5 isoforms do not have innate activity and can only form heterodimers with ERβ1 to modulate its activity [15]. ERα, ERβ and their isoforms display distinct tissue distributions and signalling responses. The isoforms differ in their impact on oestrogen signalling and target gene regulation [16]. Although the majority of isoforms work together to promote E2 signalling, some ERβ isoforms act as inhibitors impeding ERα signalling [17,18]. The graphical representation of the primary structure of the two full length ERs and their most referred isoforms is shown in Figure 1.

### Transcriptional (genomic) signalling

Oestrogen signalling can be classified into two major categories: transcriptional and non-transcriptional signalling. The classical transcriptional pathway results in modulation of gene transcription, and the non-classical pathway triggers signal transduction cascades and changes in phosphorylation. During transcriptional signalling, ERs act as transcription factors. Upon binding to E2 they undergo a conformational change, which enables receptor dimerization and translocation to the nucleus. Receptor dimers bind to the ERE located in or near promoters of target genes and trigger recruitment of co-regulators that facilitate the action of RNA polymerase II machinery, promoting gene expression. There are over 70000 EREs in the human genome, out of which 17000 are located within 15 kb of mRNA start sites [19]. The sequence of the ERE affects the binding affinity of ER, and therefore can affect in the extent of gene activation by a particular ER type/isoform [20].

E2 can also influence expression of genes that do not harbour EREs in their promoter region by indirect transcriptional signalling. In this case, instead of binding to DNA directly, they form protein–protein interactions with partner transcription factors. Examples of this signalling include but are not limited to association with FBJ murine osteosarcoma viral oncogene homologue (FOS), jun proto-oncogene (JUN), nuclear factor κB (NFκB), GATA binding protein 1 (GATA1) and signal transducer and activator of transcription 5 (STAT5) [21].

### Non-transcriptional (non-genomic) signalling

The rapid effects of E2 are mediated through non-transcriptional signalling. The receptors involved in this type of signalling are the G-protein-coupled oestrogen receptor 1 (GPER1) and certain variants of ERα and ERβ [22,23]. GPER1 is a seven trans-membrane domain G-protein-coupled receptor. Its function as an ER is still under dispute with some reports showing E2-mediated signalling, whereas others do not [23–26]. GPER1 is expressed in a number of tissues including skeletal and cardiac muscle [27].

**Figure 2:**
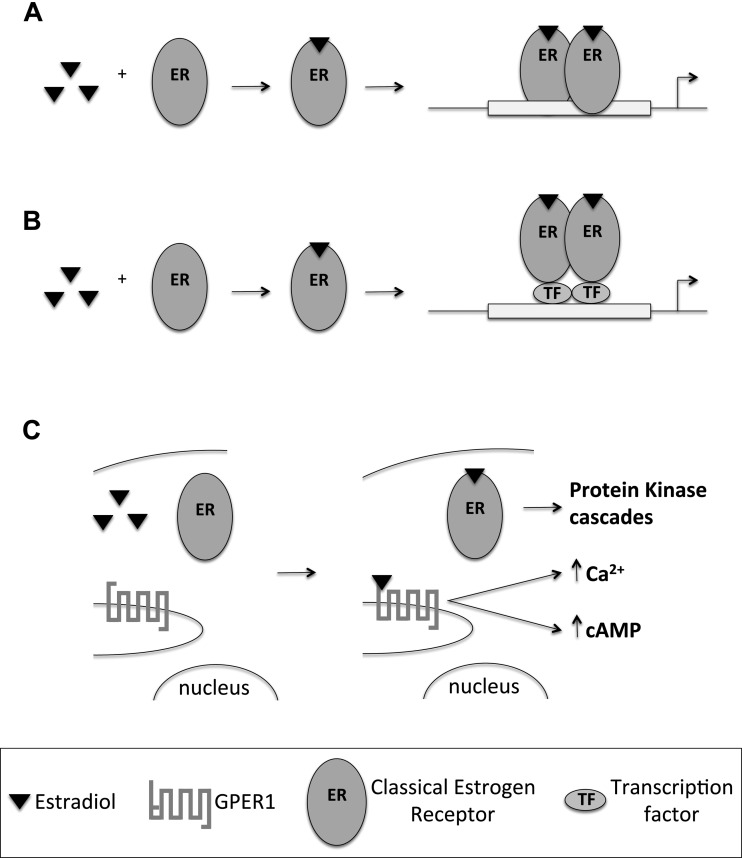
Transcriptional and non-transcriptional pathways of E2 (**A**) The direct transcriptional pathway involves interaction of ER dimers with EREs within the DNA sequence to modulate gene regulation. (**B**) The indirect transcriptional pathway involves protein–protein interaction of the ER dimers with transcription factors (TF) to regulate gene transcription. (**C**) The non-transcriptional pathway involves a subclass of classical ERs and GPER1 to trigger signal transduction cascades in response to E2 stimulation.

Some investigators suggest that a subpopulation of classical ERs reside near the cell membrane, and upon E2 stimulation form dimers that activate downstream protein cascades [28]. In contrast with GPER1, classical ERs do not contain a trans-membrane domain in their structure. Their ability to associate with the plasma membrane could be facilitated by palmoitoylation of the receptor, which promotes association with calveolin-1 [29,30]. Non-transcriptional signalling usually involves direct association of ERs with target proteins in response to E2 stimulation. This leads to activation of kinases, phosphatases and increases in ion fluxes across membranes. Examples of non-transcriptional ER signalling include mobilization of intracellular calcium, stimulation of adenylate cyclase activity and cyclic AMP (cAMP) production, activation of MAP kinase, phosphoinositol (PI) 3-kinase (PI3K) and AMP-activated protein kinase (AMPK) signalling pathways [21,31]. A schematic representation of transcriptional and non-transcriptional signalling pathways is presented in Figure 2.

From a broader perspective, both transcriptional and non-transcriptional activity of ER may affect gene expression. Whereas genomic cascades directly target gene expression, non-genomic pathways initiate signalling cascades ultimately leading to regulation of gene transcription. In other words, transcriptional and non-transcriptional signalling converge resulting in finely tuned regulation of target gene activity. One example of that is the interaction between E2/ERα and IGF-IR (insulin-like growth factor 1 receptor)/MAPK pathways. In addition to directly mediating IGF-I transcription by binding to ERE, ERα associates with IGF-I membrane receptor (IGF-IR) and activates MAP kinase cascades that influence ERα mediated gene transcription [32]. Another example of dual transcriptional and non-transcriptional action is the transcription of low-density lipoprotein receptor (LDL-R). Although the LDL-R promoter does not contain ERE, ERα interacts with the Sp1 (trans-acting transcription factor 1) transcription factor activating LDL-R gene expression [33]. In addition, tyrosine kinase activity, induced by non-transcriptional activity of E2, is required for the induction of LDL-R expression [34].

## THE DUAL ROLE OF OESTROGEN SIGNALLING IN BREAST CANCER

Breast cancer can be classified based on expression levels of ER. Breast cancer patients positive for the ER exhibit a high response rate to endocrine therapy and significantly improved prognosis over time due to advances in adjuvant therapies. Oestrogen signalling in breast cancer is complex and involves modulation of expression and activity for many different targets, ultimately favouring or counteracting cancer progression. A genome study in MCF-7 cells revealed that oestrogen activation of target genes is time dependent; 628 differentially expressed genes show a robust pattern of regulation at 12 h, 852 at 24 h and 880 differentially regulated genes at 48 h after E2 stimulation. Interestingly the majority of genes are activated at one time point, but not the other [35]. This highlights the diversity of genes and metabolic pathways that E2 affects in breast cancer, and the potential complexity and combinatorial interplay.

### Oestrogen promotes cancer growth

Some of the signalling pathways regulated by oestrogen worsen the progression of ER positive tumours. At the level of E2 transcriptional signalling in breast cancer, the transcription factor Ets1 (v-ets avian erythroblastosis virus E26 oncogene homologue 1) plays a critical role. It forms a complex with ERα and the p160 nuclear receptor coactivator family leading to the expression of ERα target genes in MCF-7 cells, promoting E2-induced tumour growth [36]. Another transcriptional target of E2/ERα signalling is the adenosine A1 receptor (Adora1). Adora1 is required for full transcriptional activity of ERα and supports breast cancer growth [37]. Among E2 targets is the pro-apoptotic protein prostate apoptosis response 4 (PAR-4). E2 decreases PAR-4 expression in breast cancer cells, providing selective advantage for breast cancer cell survival [38].

Some other transcriptionally regulated targets of E2 that induce breast cancer cell proliferation are hes family bHLH transcription factor 6 (Hes-6), prostaglandin E synthase (PTGES), alkaline phosphatases (ALP) and the LRP16 gene, just to mention a few [39–42]. Among E2 non-transcriptional signalling pathways are ERα-dependent activation of the PI3K/protein kinase B (Akt) axis [43] and ER-independent activation of maxi-K channels, both of which promote breast cancer cell growth [44]. The regulation of some oestrogen targets is more complex and, results from interplay of several transcriptional and non-transcriptional mechanisms. Examples of those targets include ERα dependent regulation of extracellular matrix molecules, repression of VEGFR2 (vascular endothelial growth factor receptor 2) mRNA levels and modulation of RIZ1 (retinoblastoma protein-interacting zinc-finger gene) and Cap43 gene expression [45–48].

Oestrogen plays an important role not only in the initiation and proliferation of breast cancers, but also cancer metastasis. A recent study suggested that up-regulation of myocardin-related transcription factor A (MRTF-A) by E2 might be a switch between proliferation-promoting and metastasis-promoting functions of E2 in ER positive breast cancer cells [49]. One of the key components in tumour metastasis is the actin-binding protein ezrin. It is frequently overexpressed in human metastatic breast cancers [50]. A recent study showed that E2 promotes breast cancer motility by phosphorylation of ezrin [51]. Another proposed mechanism leading to E2 induced metastasis is linked to E2 capacity to promote tight junction disruption during tumour progression, increasing cell motility [52]. E2 is also been linked to the functionality of p53. Loss of p53 function in breast cancer contributes to that metastatic potential of E2-responsive tumours through uncontrolled expression of the focal adhesion kinase (FAK) following E2 stimulation [53].

**Figure 3:**
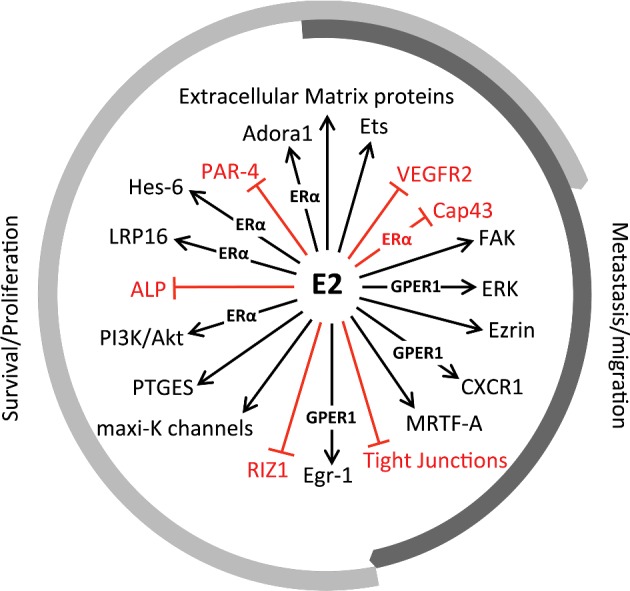
Mediators of oestrogen oncogenic effects A diagram of oestrogen targeted effectors, discussed in this review, that mediate its oncogenic effects leading to proliferation, metastasis or both. Wherever known, the involvement of ERα or GPER1 is indicated.

Not all E2 effects on cancer cells are mediated through the classical ERs. The membrane bound GPER1 also mediates some of oestrogen signalling in ER positive and ER negative cells. This explains why E2 can induce metastasis in ER negative cells *in vitro* as well as in mice [54]. E2 promotes migration and invasion in ER negative cancer by cross-talk between GPER1 and CXC receptor-1 (CXCR1), an active regulator in cancer metastasis upon binding interleukin 8 (IL-8) [55]. Non-transcriptional E2 stimulation of GPER1 in ER negative cancer cells also activates the extracellular-signal-regulated kinase (ERK) pathway, which promotes cell viability and motility [56], and increases expression of early growth response protein 1 (Egr-1) leading to transcription of genes involved in cell proliferation [57]. A diagram illustrating the oncogenic mediators of oestrogen signalling discussed above is presented in Figure 3.

Oestrogen signalling within cancer cells induces the synthesis of more E2 to fulfil the needs of the tumour by regulating key enzymes involved in oestrogen biosynthesis. Rapid non-transcriptional actions of E2 stimulate aromatase phosphorylation in breast cancer cells enhancing its enzymatic activity [58]. E2 also increases hydroxysteroid (17-beta) dehydrogenase 7 (HSD17B7) transcriptional activity, an enzyme that converts E1 to E2. This ERα dependent local synthesis of E2 instigates growth of oestrogen-dependent breast cancers [59]. Another regulator of oestrogen metabolism within cancer cells is the pro-inflammatory cytokine tumour necrosis factor alpha (TNFα). Stimulation of breast cancer cells with TNFα can lead to decreased E1/E2 ratio, by altering the expression of genes and enzymes involved in E2 activation [60]. In addition to infiltrated immune cells, ER positive breast cancer cells also secrete TNFα, in response to E2 regulation, creating a positive feedback loop for E2 synthesis [61].

### Beneficial effects of oestrogen signalling

The cellular response to E2 stimulation not always leads to cancer progression and in some cases may be beneficial. E2 signalling can impart low invasive behaviour in ERα positive breast cancer. For example, overexpression of GD3 synthase (GD3S) enhances proliferation and migration of ERα negative breast cancer cells [62]. In ERα positive tumours, E2 blocks its expression by preventing NFκB from binding to the GD3S gene ST8SIA1 (ST8 alpha-*N*-acetyl-neuraminide alpha-2,8-sialyltransferase 1) core promoter [63]. E2 can also activate PAX2 (paired box 2), a transcription factor that inhibits the expression of ERBB2 (erythroblastic leukaemia viral oncogene homologue 2), a pro-invasive and pro-metastatic gene [64]. Another way to alter invasiveness is through modification of extracellular matrix composition. ERα protects MCF-7 cells from changes in expression of extracellular matrix effectors (specifically matrix-degrading enzymes), which would otherwise lead to cell migration and invasion [65]. In addition, transcriptional signalling of E2 through ERα increases the expression of integrin α5β1, conferring a stationary status to cancer cells [66]. Breast cancer prognosis can also be improved through E2 transcriptional regulation of the PHLDA1 (pleckstrin homologue-like domain, family A, member 1) and STEAP1 (six transmembrane epithelial antigen of the prostate 1) genes [67,68].

E2 signalling is also linked to apoptosis in breast cancer cells. AMPK mediates E2-induced apoptosis in long-term oestrogen-deprived breast cancer cells [69]. c-Jun N-terminal kinase (JNK) signalling mediates the apoptotic effects of E2 at high concentrations in ERα positive but not ERα negative breast cancer cells [70]. One of the critical steps in cancer progression is the creation of new blood vessels that supply the tumour with nutrients, known as angiogenesis. A recent study showed that the expression of a known promoter of angiogenesis, angiopoietin-1 (Ang-1), is reduced by E2 in an ERα dependent manner [71].

**Figure 4:**
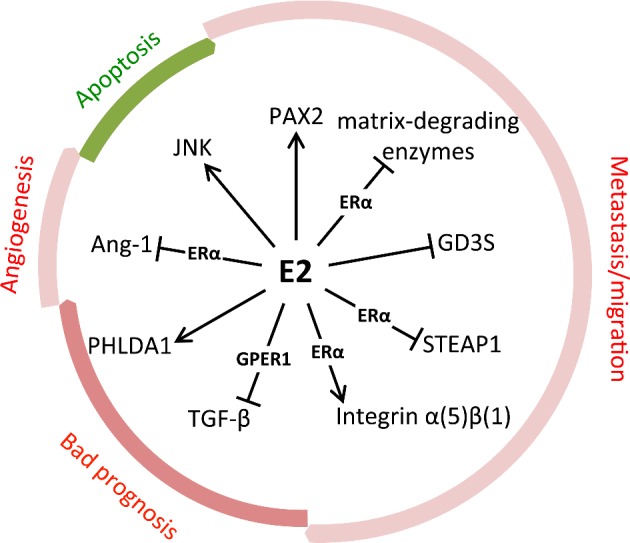
Molecular mediators of anti-tumorigenic oestrogen signalling A diagram of oestrogen targeted effectors, discussed in this review, that mediate its apoptotic, anti-metastatic, anti-angiogenic effects, or improve bad prognosis. Processes shown in green are potentiated, whereas processes shown in red are inhibited. Wherever known, the involvement of ERα or GPER1 is indicated.

As mentioned before, not all E2 signalling is ER-dependent. A study in MCF-7 cells showed that E2 can disrupt transforming growth factor beta (TGF-β) signalling by non-transcriptional activation of the GPER1 receptor, potentially involving stimulation of mitogen activated protein kinases (MAPKs) [72]. The role of TGF-β in cancer is controversial, but high levels of TGF-β correlate with poor cancer outcome [73]. A diagram illustrating the mediators of beneficial oestrogen signalling in breast cancer is presented in Figure 4.

### Determinants of oestrogen signalling outcome

The wide range of effects of oestrogen signalling during breast cancer progression may be key to the large diversity of cancer outcomes and opens up the question of what determines the nature of E2 signalling in each particular case. There are many factors that can influence the way cells react to E2 stimulation. One of these determinants is the nature of the intracellular pool of accessory molecules involved in targeted gene expression. A recent study in breast cancer T47D cells showed that the differential effects of E2/ERα signalling are dictated by recruitment of co-activators and co-repressors at target gene promoters, which is influenced by their expression levels [74].

Phosphorylation status of ERα also plays a role in determining the nature of E2 response. ERα is phosphorylated on multiple amino acid residues by several kinases in response to E2 binding. In general, phosphorylation of serine residues appears to influence the recruitment of co-activators, enhancing ER-mediated transcription [75]. Inhibition of ERα phosphorylation at Ser^118^ and Ser^167^ promotes increased growth, migration, invasion and disruption of E2 signalling in MCF-7 cells [76]. In contrast, tyrosine phosphorylation of ERα by Src regulates cytoplasmic localization of this receptor. Inhibition of Tyr^537^ phosphorylation traps ERα in the nuclei of E2 treated MCF-7 cells, and induces cell cycle arrest [77].

ERα isoform expression is a critical determinant in the assessment of breast cancer prognosis in both ER positive and ER negative tumours. ERα-36, the truncated variant of ERα-66, is expressed in both ER positive and ER negative breast cancer tumours [78,79]. It mediates non-transcriptional oestrogen signalling, resulting in prevention of apoptosis and increased growth of ER negative breast cancer cells [80,81]. It also inhibits genomic signalling by ERα-66 and ERβ [82]. Similarly, ERα-46 over-expression in endocrine treatment-resistant breast cancer cells selectively inhibits the ERα-66 response to oestrogen [83]. Therefore, a careful assessment of receptor isoform expression is important in predicting oestrogen signalling outcome.

Although more is known about signalling through ERα than ERβ, the role of the latter cannot be dismissed. Overall ERβ levels in breast cancer cell lines are lower compared with normal breast epithelium [84]. Isoform expression varies largely, ERβ4 showing higher expression in breast tumours, and ERβ3 generally absent [85]. The exact contribution of ERβ to breast cancer biology remains largely unexplored. Up to this point, ERβ has been shown to reduce cell proliferation [86,87], invasion [88,89] and angiogenesis [90]. More importantly, the ERα/β ratio within the cells influences the nature of oestrogen signalling. ER subtype ratio has been shown to regulate the effect of E2 on mitochondria proliferation, functionality and oxidative stress in breast cancer cells, in such a way that by altering the ERα/β relative expression, completely opposite outcomes of E2 signalling can be achieved [91–93]. In addition, these two receptors can influence each other activities in some cases showing antagonism [94,95]. This modulation of ERα activity could be achieved by formation of heterodimers with ERβ [96].

Lastly, E2 metabolism by cytochrome P450 enzymes can influence the fate of oestrogen signalling [97]. Within the cell E2 can be metabolized giving rise to different molecules. Among these are some metabolites like 2-hydroxyoestradiol (2-OHE2) and 4-hydroxyoestradiol (4-OHE2) that promote tumorigenesis by increasing cell proliferation and formation of reactive oxygen species known to instigate DNA mutations [98,99]. Other metabolites, like 2-metoxyoestradiol (2-ME) have the opposite effect on cancer promoting apoptosis of tumour cells [100].

## FINAL REMARKS

Taken together, all the information presented above suggests that E2 signalling in breast cancer is very complex and cannot be categorized as detrimental or beneficial without prior knowledge of function. It is the particular combination of molecular assets within the cancer cell that helps fine-tune the course of molecular events triggered by oestrogen. The overall response to oestrogen stimulation can be modulated at different levels within the cells. These regulatory levels can be classified as receptor-dependent or receptor-independent. The first one refers to ER expression status, presence of post-translational modifications and formation of functional dimers. The latter includes alternative metabolic processing of oestrogen and the unique pool of intracellular effectors that can be found in each cell type.

The next question is how can we translate the current state of the literature into specialized treatment options. A large portion of treatments available for breast cancer act by blocking oestrogen synthesis and/or signalling, therefore, preventing oestrogen-induced tumour proliferation. Unfortunately, besides not taking into account the protective oestrogen effects, they are highly non-specific, possess adverse side effects and can result in endocrine resistance [101,102]. On the other end, ER agonists have also demonstrated clinical efficacy in breast cancer treatment [103]. Their use is not as common, since they are only employed as alternative to advanced cancers showing resistance to anti-oestrogen treatment [104,105].

It is likely that the contradictory findings on the cellular and molecular impact of oestrogen in the literature are rooted in the dual effects of oestrogen signalling. Consequently, a reassessment of the literature may reveal that oestrogen treatment can be considered as an alternative cancer therapy. Because many of the conclusions regarding the therapeutic use of oestrogen are drawn from clinical trials, more mechanistic studies will better exploit or predict therapeutic strategies to selectively potentiate the anti-cancer effects of oestrogen signalling and create specialized treatment regimens for breast cancer patients.

## Abbreviations

Adora1: adenosine A1 receptor
AF: activation function
Akt: protein kinase B
ALP: alkaline phosphatases
AMPK: AMP-activated protein kinase
Ang-1: angiopoietin-1
CXCR1: CXC receptor-1
DBD: DNA-binding domain
E1: oestrone
E2: 17β-oestradiol
E3: oestriol
Egr-1: early growth response protein 1
ERα: oestrogen receptor alpha
ERβ: oestrogen receptor beta
ERBB2: erythroblastic leukaemia viral oncogene homologue 2
ERE: oestrogen response element
ERK: extracellular-signal-regulated kinase
Ets1: v-ets avian erythroblastosis virus E26 oncogene homologue 1
FAK: focal adhesion kinase
FOS: FBJ murine osteosarcoma viral oncogene homologue
GATA1: GATA binding protein 1
GD3S: GD3 synthase
GPER1: G-protein-coupled oestrogen receptor 1. Hes-6: hes family bHLH transcription factor 6
HSD17B7: hydroxysteroid (17-beta) dehydrogenase 7
IGF-IR: insulin-like growth factor 1 receptor
JNK: c-Jun N-terminal kinase
LBD: ligand-binding domain
LDL-R: low-density lipoprotein receptor
MAPK: mitogen activated protein kinase
2-ME: 2-metoxyoestradiol
MRTF-A: myocardin-related transcription factor A
NFκB: nuclear factor κB
NTD: N-terminal domain
2-OHE2: 2-hydroxyoestradiol
4-OHE2: 4-hydroxyoestradiol
PAR-4: prostate apoptosis response 4
PAX2: paired box 2
PHLDA1: pleckstrin homologue-like domain, family A, member 1
PI3K: phosphoinositol (PI) 3-kinase
PTGES: prostaglandin E synthase
RIZ1: retinoblastoma protein-interacting zinc-finger gene
Sp1: trans-acting transcription factor 1
STAT5: signal transducer and activator of transcription 5
STEAP1: six transmembrane epithelial antigen of the prostate 1
TGF-β: transforming growth factor beta
TNFα: tumour necrosis factor alpha
VEGFR2: vascular endothelial growth factor receptor 2
ST8SIA1: ST8 alpha-*N*-acetyl-neuraminide alpha-2,8-sialyltransferase 1
